# Accuracy of fluoroscopic examination in the treatment of Bennett’s fracture

**DOI:** 10.1186/s12891-020-03867-1

**Published:** 2021-01-04

**Authors:** Yaobin Yin, Yanqing Wang, Zhilong Wang, Wenrui Qu, Wen Tian, Shanlin Chen

**Affiliations:** Department of Hand Surgery, Beijing Ji Shui Tan Hospital and the 4th Medical College of Peking University, Xin jie kou dong jie 31, Xi Cheng Qu, Beijing, 100035 China

**Keywords:** Bennett’s fracture, Fluoroscopy, Gap, Reduction, Step-off

## Abstract

**Background:**

Restoration of joint congruity is an important factor for the prevention of subsequent arthritis in patients with Bennett’s fracture. Surgical treatment of Bennett’s fracture is thus generally recommended for displaced intra-articular fractures to the proximal aspect of the thumb metacarpal. Fluoroscopic examination is used to evaluate the adequacy of closed reduction after pinning of Bennett’s fracture. The purpose of this study was to determine the accuracy of fluoroscopy to determine the reduction of Bennett’s fractures.

**Methods:**

A model was created, to mimic a Bennett’s fracture utilizing ten fresh-frozen cadaveric hands. An oblique cut was made in the proximal aspect of the thumb metacarpal using an oscillating saw. The small oblique fragment involved 1/4–1/3 of the joint surface was then shifted in position creating a step-off or gap at the fracture site. An anatomical reduction model, gap models (1 mm, 2 mm, 3 mm), and step-off models (1 mm, 2 mm, 3 mm) were created using percutaneous fixation with two 1.0 mm Kirschner wires for each cadaveric hand. Fluoroscopic assessment then took place and was reviewed by 2 attending hand surgeons blinded to the actual position. Their estimated fluoroscopic position was then compared to the actual displacement.

**Results:**

The step-off and gap on fluoroscopic examination showed a significant difference compared to the step-off and gap from direct visualization. The frequency of underestimation for the 3 mm displacement models from the fluoroscopic examination was 60%. The frequency for overestimated was 9% for the models in which displacement was within 2 mm (0, 1, 2 mm).

**Conclusions:**

The assessment of articular gap and step-off using PA (postero-anterior), AP (antero-posterior), and lateral view of fluoroscopic examination is not accurate as compared to the examination by direct visualization. Surgeons need to be aware that PA, AP and lateral view of fluoroscopic examination alone may not be sufficient to judge the final position of a reduced Bennett’s fracture. Other methods such as live fluoroscopy in multiple different planes, 3-dimensional fluoroscopy or arthroscopic examination should be considered.

## Background

The Bennett’s fracture is an intra-articular injury that occurs at the proximal aspect of the thumb metacarpal, and is the most common fracture involving the carpometacarpal joints. Zhang et al. [1] reported that Bennett’s fracture accounted for around 12% of metacarpal fractures and 2% of all hand fractures. A non-anatomic reduction(joint surface displacement>2 mm) and malunion of Bennett’s fracture can lead to joint subluxation and arthritis [2]. It is for this reason that surgical management is generally recommended for displaced intra-articular fractures to the proximal aspect of the thumb metacarpal. Closed reduction and percutaneous fixation is most widely used method for Bennett’s fracture [3–6]. Following closed reduction and percutaneous fixation, fluoroscopic imaging can be used to assess the reduction. However, there are conflicting views in the literature as to the accuracy of fluoroscopic assessment following proximal thumb metacarpal fractures reduction [7, 8]. The purpose of this study was to determine the accuracy of fluoroscopy to determine the reduction of Bennett’s fractures.

## Methods

**Fig. 1:**
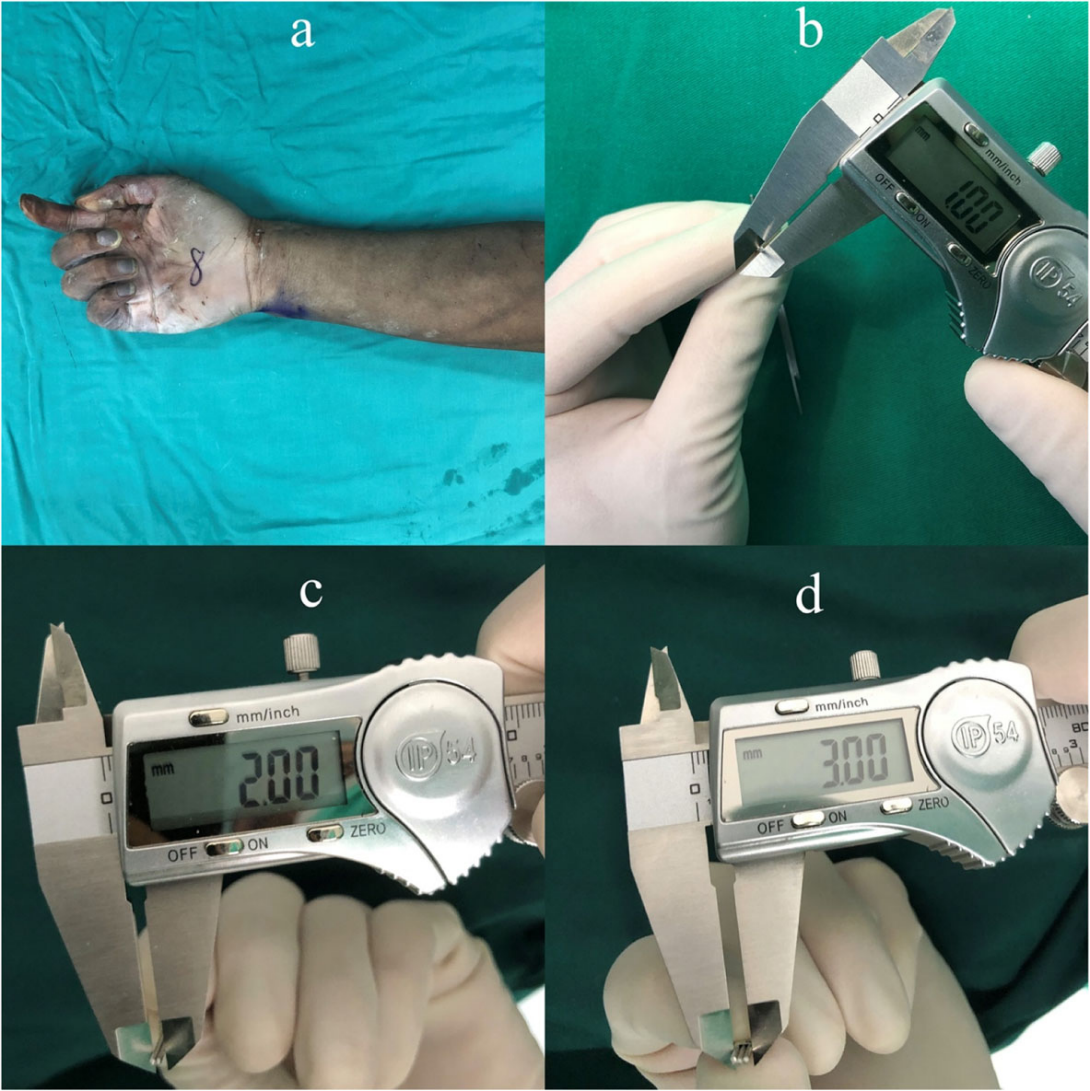
**a**: fresh-frozen preserved cadaver forearm was used; **b**-**d** 1 mm Kirschner wires were used to judge the displacement of fractures from 1 mm to 3 mm

**Fig. 2:**
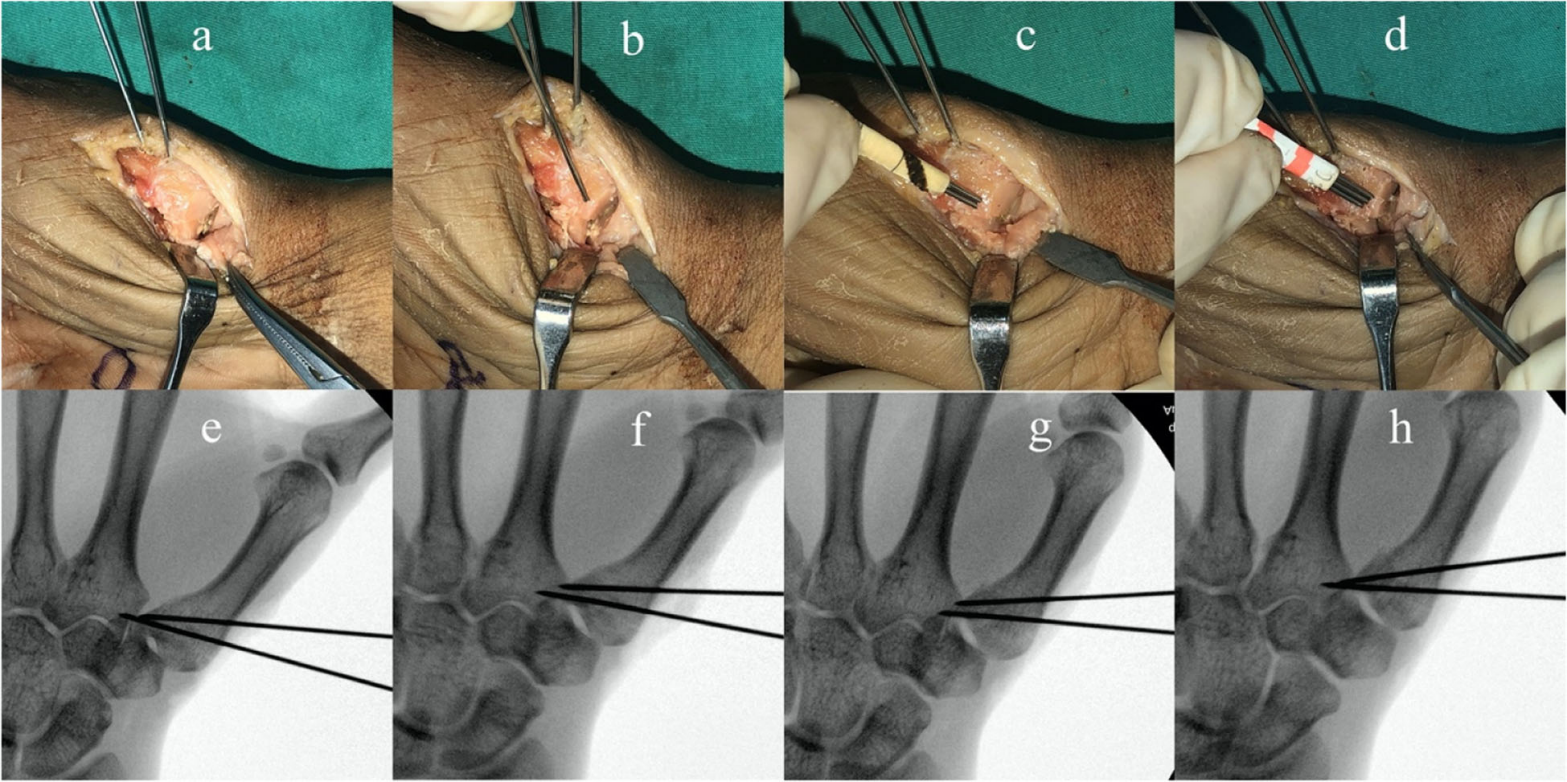
**a**: anatomy reduction models; **b**:1 mm step-off model; **c**: 2 mm step-off model; **d**: 3 mm step-off model; **e**: fluoroscopy image of (**a**); **f**: fluoroscopy image of (**b**); **g** fluoroscopy image of (**c**); **h**: fluoroscopy image of (**d**)

**Fig. 3:**
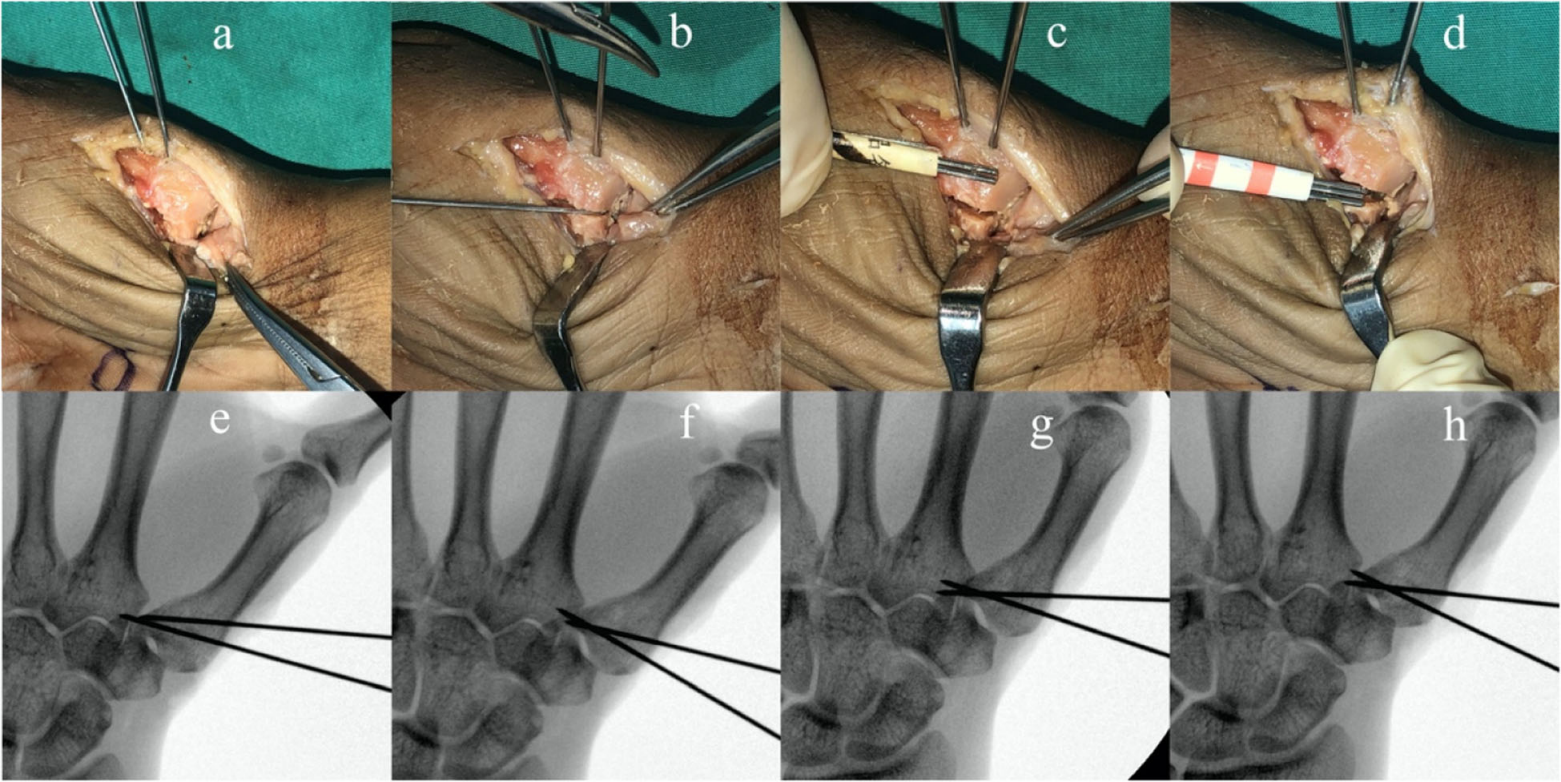
**a**: anatomy reduction model; **b**: 1 mm gap model; **c**: 2 mm gap model; **d**: 3 mm gap model; **e**: fluoroscopy image of (**a**); **f**: fluoroscopy image of (**b**); **g** fluoroscopy image of (**c**); **h**: fluoroscopy image of (**d**)

The cadaver forearms used in this study were offered by Department of Anatomy of Peking University. The use of specimens for scientific investigations is in accordance with the Declaration of Helsinki and was approved by the ethical committee in our hospital. On the basis of previous study [7, 8], the calculated samples sized was 10 for 80% power to detect a difference of 1 mm for the displacement with a two-sided significance level of 0.05. A total of ten fresh-frozen preserved cadaver forearm (five right and five left) were used. None of them had bony deformities or lesions checked by fluoroscopy. The Bennett’s fractures were created using an open Wagner approach [9]. The thenar muscles were stripped extraperiosteally and reflected from the volar aspect of the trapezium and metacarpal bone. A longitudinal incision of the capsule was made to expose the proximal aspect of the thumb metacarpal. Bennett’s fractures were created by an oscillating saw. All osteotomies were performed with an oscillating saw at an angle of 20°-30° to the thumb metacarpal, which resulted in an intra-articular fracture at the proximal aspect of the thumb metacarpal with only one intra-articular fragment at the ulnar aspect. The osteotomy plane was in line with the lateral view of the thumb metacarpal. The fracture involved approximately 1/4–1/3 of the joint surface measured from volar to dorsal. For each cadaveric hand, an anatomical reduction model, and displacement models involving 2 separate aspect were created, one mimicking a gap and the second mimicking a step-off. The small separated fragment was carefully positioned and measured by one to three 1 mm K wires confirmed using a measuring caliper (band name: Biaokang, precision: 0.01 mm) (Fig. 1). The small separated fragment was positioned with sequentially, either a step-off or gap of 1 mm, 2 mm, and 3 mm and held with two K-wires in each instance. Fluoroscopic images were taken in 3 separate planes, postero-anterior (PA), antero-posterior (AP), and lateral (Figs. 2, 3). Double check for the displacement of the fracture by one to three 1 mm Kirschner wires before and after getting fluoroscopic images. Two different hand surgery attendings who did not know the models, on two occasions, recorded the degree of displacement in terms of step-off or gap. A digital image program (Image J; Scion Corp, Frederick, Maryland, USA) was used to measure intra-articular step-off and gap on the fluoroscopic images These recordings with then compared to the actual degree of measured displacement.

Fracture gap and step-off were measured to the nearest 0.1 mm for the modalities (fluoroscopy, direct visualization). Step-off was defined as the largest intra-articular step perpendicular to the joint surface. Gap was defined as the largest intra-articular gap parallel to the joint surface between two fracture fragments. The results from fluoroscopic imaging measurements were compared with the results from direct visualization using paired t-tests statistical test. We also examined the differences of the two attending doctors’ results by paired t-tests statistical test. A *p* value < 0.05 was considered statistically significant. Intra-class correlation coefficients were calculated to assess the agreement between the two attending doctors’ results. The intra-class correlation is a descriptive statistic that describes how strongly the two attending doctors resemble each other for the measurement with a number range of 0–1. The guideline for the interpretation of intra-class correlation is the following: 0.7–1.0 as good; 0.4–0.69 as medium; 0–0.39 as poor. The frequency of overestimate and underestimate was calculated. 2 mm displacement was threshold value for reduction. Overestimate in this study was defined as the measurement result for fluoroscopy images for models (0, 1, 2 mm gap or step-off) was more than 2 mm. Underestimate in this study was defined as the measurement result for fluoroscopy images for models (3 mm gap or step-off) was less than 2 mm.

## Results

There were seven models (anatomy reduction models, gap models (1 mm, 2 mm, 3 mm) and step-off models (1 mm, 2 mm, 3 mm)) for each cadaveric hand and a total of 210 fluoroscopic images (70 PA views, 70 AP views, and 70 lateral views) were obtained. In all PA and AP views, displacement of the joint surface could not be observed. Two attending doctors who did not know the detail of the models measured the displacement (step-off and gap) of the joint surface on 70 lateral images independently. The results of two attending doctors were shown in Table 1. There was no significant difference between two attending doctors’ results using paired t-tests statistical test (*P* > 0.05). The agreement between the two attending doctors was good for the step-off (intra-class correlation coefficient 0.78) and medium for the gap (intra-class correlation coefficient 0.44).

**Table 1:** Measurements of step-off and gap between 1st and 2nd attending doctor

	Sample size	Step-off			Gap		
		Mean (mm)	SD (mm)	*P* value	Mean (mm)	SD (mm)	*P* value
The 1st Attending doctor	70	0.9	0.9		1.1	0.7	
The 2nd Attending doctor	70	1.0	1.1		0.9	1.1	
				0.20			0.07

On fluoroscopic images, the mean step-off value was 1.1 mm (SD 0.9) and the mean gap value was 1.0 mm (SD 0.8). Significant difference was noted for the measurements obtained from fluoroscopic examination versus open visualization for step-off (*p* < 0.05) and gap (p < 0.05). For specific models, all the models show significant difference except for the model with 1.00 mm displacement (Table 2). The displacement was underestimated by fluoroscopic image in most conditions, especially for the 3 mm displacement models. The frequency for underestimated for the 3 mm displacement models was 60% (two attending doctors, 55 and 65% respectively). The frequency for overestimated was 9% (two attending doctors, 7 and 12% respectively) for models which displacement was within 2 mm (0, 1, 2 mm).

**Table 2:** Measurements of different displacement fracture models by fluoroscopic imaging and direct visualization

Displacement	Measurement method	Sample size	Mean (mm)	SD (mm)	*P* value
0 mm	Fluoroscopic imaging	20	0.5	0.5	
	Direct visualization	20	0.0	0.0	
					0.00*
1 mm	Fluoroscopic imaging	20	0.9	0.6	
	Direct visualization	20	1.0	0.0	
					0.60
2 mm	Fluoroscopic imaging	20	1.2	0.8	
	Direct visualization	20	2.0	0.0	
					0.00*
3 mm	Fluoroscopic imaging	20	1.6	1.0	
	Direct visualization	20	3.0	0.0	
					0.00*

*P < 0.05

## Discussion

### The development of treatment of Bennett’s fracture

The Bennett’s fracture is an intra-articular injury that occurs at the proximal aspect of the thumb metacarpal. The pattern of injury was first described by Edward H. Bennett’s at the University of Dublin in 1882 [10]. In 1952 at Sahlgren’s Hospital at the University of Goteborg, Sweden, Moberg, and Gedda described open reduction and internal fixation of Bennett’s fractures [11]. Closed reduction and casting remained the preferred method of treatment until the 1970s. Although historical reports have noted satisfactory outcomes with nonsurgical treatment, more recent studies have shown poor outcomes with casting alone for this injury [12–15]. Surgical treatment includes closed reduction with percutaneous pinning or open reduction with either pins or inter fragmentary fixation [16]. With the development of arthroscopy, some doctors prefer arthroscopic reduction and internal fixation [17, 18]. There are various methods of fixation: pins [3–6, 19], screws [17, 20], plates [9, 21], tension bands [22, 23], and external fixator [24]. Most researchers believed that anatomic congruity is more important than fracture fixation technique for long-term success. The exact degree of persistent joint surface displacement that leads to long-term symptoms is still controversial. Kjaer-Petersen and colleagues [12] reported that the quality of the reduction was highly related to long-term symptoms. Kamphuis [2] found that a persistent step-off or gap larger than 2 mm after surgical fixation was correlated with post-traumatic arthritis at 10 years’ follow-up and they concluded that Bennett’s fractures could be safely treated when the persistent step-off and gap do not exceed 2 mm after the fixation.

### Accuracy of fluoroscopy for judging the reduction of Bennett’s fracture

Closed reduction and percutaneous fixation of Bennett’s fracture were recommended by most surgeons [4, 25]. Following closed reduction and percutaneous fixation, fluoroscopic imaging can be used to assess the reduction. Capo et al. [7] created Bennett’s fractures and closed the incision in eight fresh-frozen cadaveric hands. Under fluoroscopic visualization, the fractures were treated by closed reduction and pinned using 1.14-mm (0.045-in) K-wires. These reductions were judged as being in alignment with fracture step-off and displacement less than 1.5 mm under fluoroscopic visualization. Anteroposterior and lateral plain radiographic films were used to assess the reduction. Finally, the carpometacarpal joint was opened and visualized to directly assess the reduction for fracture step-off, displacement, and gap. They found that the values for step-off and displacement were significantly different as compared to the values from the direct measurements. Another study performed by Greeven et al. [8] draw an opposite conclusion and they claimed that fluoroscopic visualization during operation provided an adequate assessment of articular step-off and displacement in comparison with radiographs and direct visualization. Greeven et al. believed that there were a few reasons for the inconsistency. The first reason was the different precision of measurements in Capo’s study. The second reason was that the models used in Capo’s study were based on the hands that were already fully dissected, which made the fracture fixation more prone to displacement during the experiment [8]. An additional reason might be due to the small sample size used in both of the previous studies. In our study, we used ten well preserved fresh-frozen cadaver forearms and all hands were intact. For each cadaveric hand, seven different reduction models were simulated consecutively. For each hand, we could get eight group data (step-off: 0 mm, 1 mm, 2 mm, 3 mm; gap: 0 mm, 1 mm, 2 mm, 3 mm) and used Image J to analyze the image. Our study could simulate the clinical reduction of Bennett’s fracture properly and have more data than previous studies. Fracture gap and step-off were measured with a precision of 0.1 mm for fluorography and open visualization. There might be some little gap in the large step-off models and some little step-off in large gap models because it was really difficult to make displacement fracture models in one direction only for large step-off or gap displacement. Direct visualization measurement of the little gap in large step-off models and the little step-off in large gap models by digital caliper is difficult and imprecise. We thus did not measure gap in step-off models and step-off in gap models.

There were two types of displacement of Bennett’s fracture used in this study. Step-off was defined as the largest intra-articular step perpendicular to the joint surface which was the most common in Bennett’s fracture. Gap was defined as the largest intra-articular gap parallel to the joint surface between two fracture fragments which was underestimated by many surgeons. It should be noted that the fracture gap is widely unreduced and there is still a gap when the hand in the hitchhiker position (in which the joint space is wide), because there is no tension on the dorsal radial ligament complex and the fracture is partially reduced by simply opposing the thumb. The gap will disappear only when the thumb metacarpal is twisted passively into the last phase of opposition [26].

**Fig. 4:**
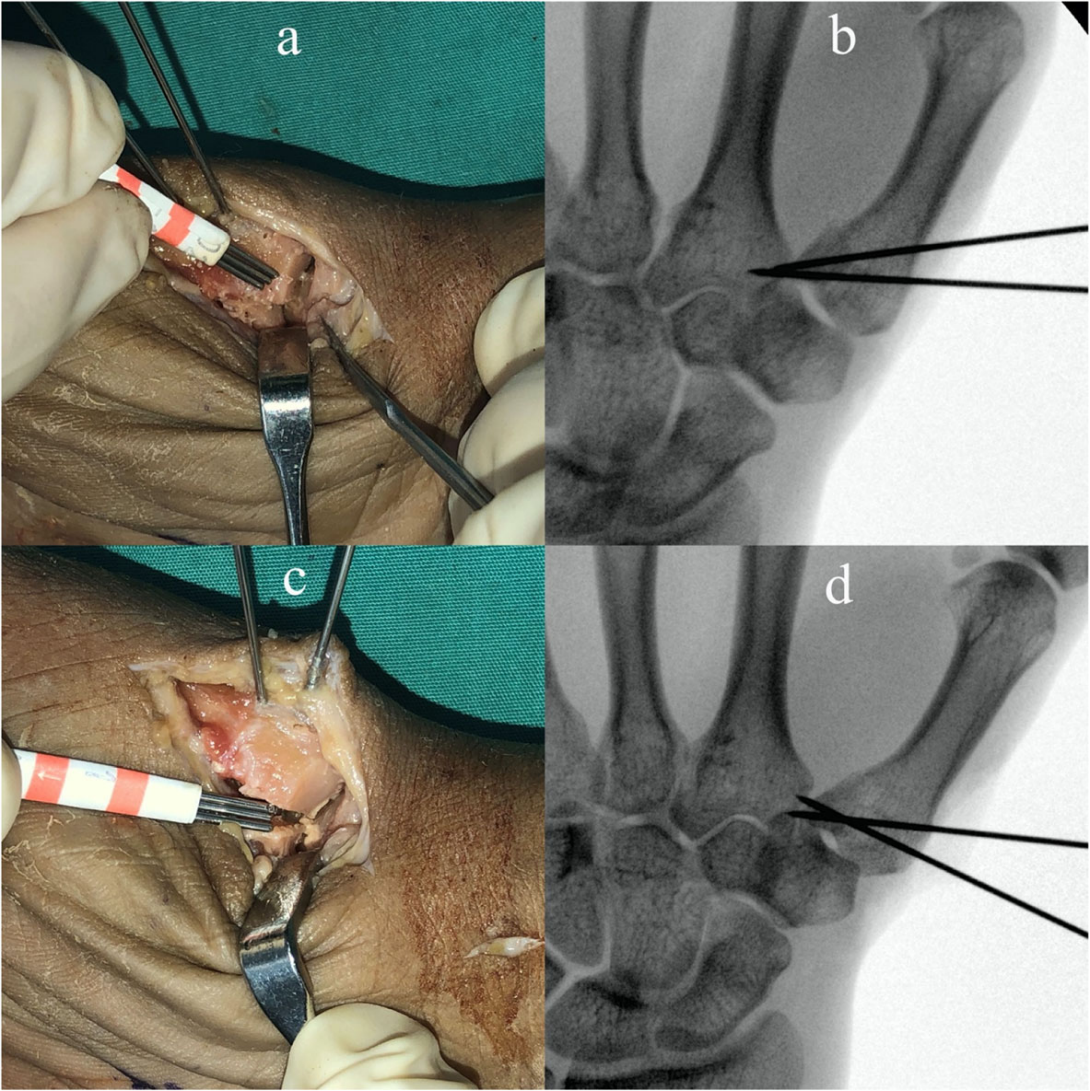
The difference between direct view and fluoroscopic view. **a**: 3 mm step-off model; **b**: fluoroscopic view of (**a**); **c**: 3 mm gap model; (**d**): fluoroscopic view of (**c**)

In the present study, we found the assessments using fluoroscopic imaging underestimated the degree of displacement when compared with direct visualization for step-off and gap. (Fig. 4). The frequency for underestimated for 3 mm models was 60% (two attending doctors, 55 and 65%). In underestimate conditions, the surgeon would not change the reduction when it should have been changed. The frequency for overestimated was 9% (two attending doctors, 7 and 12%) for models which displacement was within 2 mm (0, 1, 2 mm). In overestimate conditions, the surgeon would change the reduction when it should not have been changed. It is thus highly necessary that surgeons could not judge the reduction only by PA, AP, and the lateral view from fluorography image, and more different positions are needed.

One limitation could be kept in mind when we interpreted this study. It is known that it is much more complicated for real Bennett’s fracture in which step-off and gap always happen together, which should be treated simultaneously. However, we created the models and assessed them separately which might lead to overestimation of the accuracy of the fluoroscopic examination. The other limitation was that K-wires themselves were obscuring the reduction from accurate visualization on fluoroscopy images for some models. But in most conditions, the attending doctors could perform the measurement from the images. There was no significant difference between the two attending doctors’ results and the agreement between the two attending doctors’ results was good for the step-off (intra-class correlation coefficient 0.78) and medium for the gap (intra-class correlation coefficient 0.44).

## Conculsions

In summary, surgeons need to be aware that PA, AP and lateral view of fluoroscopic examination may not be sufficient to judge the final position of a reduced Bennett’s fracture. Other methods such as live fluoroscopy in multiple different planes, 3-dimensional fluoroscopy, or arthroscopic examination should be considered.

## Data Availability

The datasets used and/or analyzed during the current study are available from the corresponding author on reasonable request.

## Abbreviations

PA: Postero-anterior
AP: Antero-posterior

## References

[1] Yingze Z (2012). Clinical epidemiology of orthopedic trauma.

[2] Kamphuis SJM, Greeven APA, Kleinveld S, Gosens T, Van Lieshout EMM, Verhofstad MHJ (2019). Bennett's fracture: comparative study between open and closed surgical techniques. Hand Surg Rehab.

[3] Bennani A, Zizah S, Benabid M, Almoubaker S, Chbani B, Lahrach K (2012). The intermetacarpal double pinning in the surgical treatment of Bennett fracture (report of 24 cases). Chirurgie de la main.

[4] Greeven AP, Alta TD, Scholtens RE, de Heer P, van der Linden FM (2012). Closed reduction intermetacarpal Kirschner wire fixation in the treatment of unstable fractures of the base of the first metacarpal. Injury.

[5] Adi M, Miyamoto H, Taleb C, Zemirline A, Gouzou S, Facca S (2014). Percutaneous fixation of first metacarpal base fractures using locked K-wires: a series of 14 cases. Tech Hand Upper Extrem Surg.

[6] Middleton SD, McNiven N, Griffin EJ, Anakwe RE, Oliver CW (2015). Long-term patient-reported outcomes following Bennett's fractures. Bone Joint J.

[7] Capo JT, Kinchelow T, Orillaza NS, Rossy W (2009). Accuracy of fluoroscopy in closed reduction and percutaneous fixation of simulated Bennett's fracture. J Hand Surg.

[8] Greeven AP, Hammer S, Deruiter MC, Schipper IB (2013). Accuracy of fluoroscopy in the treatment of intra-articular thumb metacarpal fractures. J Hand Surg Eur Vol.

[9] Uludag S, Ataker Y, Seyahi A, Tetik O, Gudemez E (2015). Early rehabilitation after stable osteosynthesis of intra-articular fractures of the metacarpal base of the thumb. J Hand Surg Eur Vol.

[10] Bennett EH (1882). Fractures of the metacarpal bone. Dublin J Med Sci.

[11] Moberg EGKO (1952). Surgical therapy of Bennett's fracture.

[12] Kjaer-Petersen K, Langhoff O, Andersen K (1990). Bennett’s fracture. J Hand Surg (Edinburgh, Scotland).

[13] Oosterbos CJ, de Boer HH (1995). Nonoperative treatment of Bennett's fracture: a 13-year follow-up. J Orthop Trauma.

[14] Timmenga EJ, Blokhuis TJ, Maas M, Raaijmakers EL (1994). Long-term evaluation of Bennett's fracture. A comparison between open and closed reduction. J Hand Surg (Edinburgh, Scotland).

[15] Livesley PJ (1990). The conservative management of Bennett's fracture-dislocation: a 26-year follow-up. J Hand Surg (Edinburgh, Scotland).

[16] Greeven APA, Van Groningen J, Schep NWL, Van Lieshout EMM, Verhofstad MHJ (2019). Open reduction and internal fixation versus closed reduction and percutaneous fixation in the treatment of Bennett fractures: a systematic review. Injury.

[17] Pomares G, Strugarek-Lecoanet C, Dap F, Dautel G (2016). Bennett fracture: arthroscopically assisted percutaneous screw fixation versus open surgery: functional and radiological outcomes. Orthop Traumatol Surg Res.

[18] Solomon J, Culp RW (2017). Arthroscopic Management of Bennett Fracture. Hand Clin.

[19] Culp RW, Johnson JW (2010). Arthroscopically assisted percutaneous fixation of Bennett fractures. J Hand Surg.

[20] Leclère FM, Jenzer A, Hüsler R, Kiermeir D, Bignion D, Unglaub F (2012). E vg. 7-year follow-up after open reduction and internal screw fixation in Bennett fractures. Arch Orthop Trauma Surg.

[21] Pavić R, Malović M (2013). Operative treatment of Bennett's fracture. Collegium Antropologicum.

[22] Zhang X, Shao X, Zhang Z, Wen S, Sun J, Wang B (2012). Treatment of a Bennett fracture using tension band wiring. J Hand Surg.

[23] Zhang X, Dhawan V, Zhao S, Yu Y, Shao X, Zhang G (2019). Treatment of Bennett fractures with tension-band wiring through a small incision under loupes and a headlight. Phys Sportsmed.

[24] Li Z, Guo Y, Tian W, Tian G (2014). Closed reduction external fixator fixation versus open reduction internal fixation in the patients with Bennett fracture dislocation. Chin Med J.

[25] Liverneaux PA, Ichihara S, Hendriks S, Facca S, Bodin F (2015). Fractures and dislocation of the base of the thumb metacarpal. J Hand Surg Eur Vol.

[26] Edmunds JO (2006). Traumatic dislocations and instability of the trapeziometacarpal joint of the thumb. Hand Clin.

